# The Usefulness of Pine Timber (*Pinus sylvestris* L.) for the Production of Structural Elements. Part I: Evaluation of the Quality of the Pine Timber in the Bending Test

**DOI:** 10.3390/ma13183957

**Published:** 2020-09-07

**Authors:** Radosław Mirski, Dorota Dziurka, Monika Chuda-Kowalska, Marek Wieruszewski, Jakub Kawalerczyk, Adrian Trociński

**Affiliations:** 1 Department of Wood Based Materials, Faculty of Wood Technology, Poznań University of Life Sciences, Wojska Polskiego 38/42, 60-627 Poznań, Poland; marek.wieruszewski@up.poznan.pl (M.W.); jakub.kawalerczyk@up.poznan.pl (J.K.); adrian.trocinski@up.poznan.pl (A.T.); 2 Institute of Structural Analysis, Faculty of Civil and Transport Engineering, Poznan University of Technology, pl. Sklodowskiej-Curie 5, 60-965 Poznań, Poland; monika.chuda-kowalska@put.poznan.pl

**Keywords:** modulus of elasticity, pine wood, wood defects, knots, laboratory tests

## Abstract

The study assessed the quality of pine lumber by marking the modulus of elasticity in the horizontal system. The research material was a plank with the following dimensions: 137 mm wide × 39.50 mm thick × 3485 mm long. The pine wood was obtained by sawing timber in the form of logs with round cross sections and originating from the Forest Division Olesno (50°52′30″ N, 18°25′00″ E). Each long log was sawn to provide four logs of about 3.5 m, which were marked as butt-end logs (O), middle logs (S)—2 items, and top logs (W). The origin of the logs from the trunk (*Pinus sylvestris* L.) has a significant impact on the physical and mechanical properties of the wood from which they are made. Only butt-end logs (log type O) allows for the production of high-quality timber elements. The pine timber that was evaluated in this paper had a high density of about 570 kg/m^3^ and a high percentage of timber items were assigned to class C24 and higher (above 50%). The adopted horizontal model of evaluation of the modulus of elasticity gave similar results to those obtained in an evaluation according to the EN-408.

## 1. Introduction

To improve the applicability of timber in the construction industry, manufacturers are required to provide components with as low a number of defects as possible. Such components are made both as solid and glued semi-products [1,2,3]. It is the top priority to obtain dimensionally stable components with very good mechanical properties from readily available pine wood (*Pinus sylvestris* L.) originating from Polish forests [4,5,6,7,8,9,10]. Glued laminated timber has typical features of a solid timber: light weight, good strength, elasticity, durability, easy processing, and a unique feature (i.e., is readily shaped cross-sections). Its cross-section has a layered structure, enabling the manufacturing of components with variable cross-sectional height as needed [11,12,13,14].

The complete and practical application of pine wood for the manufacturing of glued components requires timber strength grading [2,15,16,17,18,19]. The procedure is based on the fundamental knowledge of the structure of wood and the defects it may have [20,21,22,23,24]. The preferred timber grading techniques are based on the use of machines in accordance with EN 338, EN 408, and EN 14081-1 [25,26,27]. If the machines are unavailable, visually graded timber is used. In Poland, the method is based on the Polish Standard PN-D-94021-10P [28].

The wood of the pine tree is valued for its high availability, good strength characteristics, and high productivity of each long log, which is due to the specific anatomical characteristics of this species. As previous studies have shown, not only the geographical region, but also the location of the log in a given tree zone influences the quality of the sawn timber harvested. It was shown that the middle and butt logs had a lower number of defects. Moreover, the proportion of raw material without defects increases as the distance from the core increases. The occurrence of a large number of knots in the Polish raw material is considered a negative quality characteristic. It is known that the number and intensity of defects affects the static bending strength of lumber.

The most frequently determined strength parameter of materials is the modulus of elasticity in static bending. The parameter is a basis for the comparison of the technical value of timber: the higher the modulus, the better the technological quality of structural materials. As the structure of the wood is anisotropic, and to ensure some flaws can be masked, the value of the modulus of elasticity does not always translate directly into proportional bending strength. This problem makes it necessary to check properties, identified in non-destructive testing, with real strength. Since the evaluation of strength requires the destruction of the tested material, it is more advantageous to determine the relationship between the modulus and the strength of the laboratory test [2,14,17].

The numerous advantages of glued laminated timber enable its use in practically every type of building regardless of its intended application. This assumption is the basis for undertaking the quality testing of glued semi-products and components, taking into consideration their use for construction purposes.

Assessing the impact of the quality of the various classes of timber originating from Polish forests to be used as sawn timber or glued components may provide valuable information to wood technology specialists in Poland, contributing to developments in the production of wood for construction applications in the form GLT (glued laminated timber).

The aim of the study was to assess the quality of pine timber in a horizontal bending test. Pieces of timber intended for testing were obtained by sawing logs from model trees from a 125-year-old stand.

## 2. Materials and Methods

**Figure 1:**
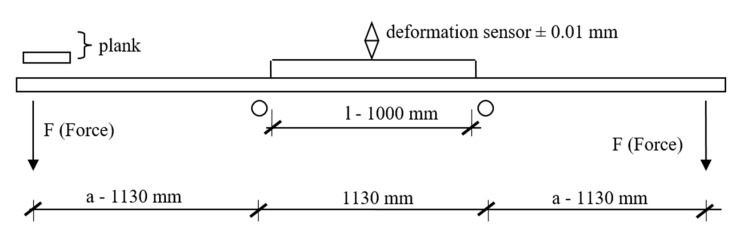
The experimental setup used to identify MOE parameter.

**Figure 2:**
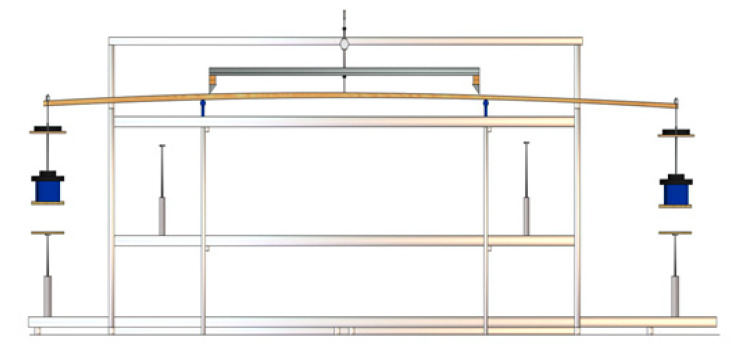
Lumber test stand (Grzegorz Zmyślony).

The research material was pine wood with the following dimensions: 137 mm wide × 39.50 mm thick × 3485 mm long. The pine wood was obtained by sawing timber in the form of logs with round cross sections and originating from the Forest Division Olesno (50°52′30″N, 18°25′00″E). The age of the tree stand was about 125 years, and the average tree height was 25 m. Butt-end long logs of about 14 m were obtained from every timber item. Each long log was sawn to provide four logs of about 3.5 m, which were marked as butt-end logs (O), middle logs (S)—2 items, and top logs (W). Each log was processed to obtain a flitch from which the main timber intended for manufacturing structural beams was obtained. Each timber item was measured to determine its linear dimensions, density, and modulus of elasticity (MOE). MOE was found by determining the deflection for a given load in accordance with the diagram shown in Figure 1. Figure 2 shows the appearance of the test stand. The assumed preliminary load was 134.9 N (13.75 kg). At that value, the deformation sensor was reset before increasing the load to 517.5 N (52.75 kg). The timber was deflected eight times, but the deflection values were recorded for the final five measurements only.

The modulus of elasticity (MOE) of lumber is calculated from Equation (1):

(1) $$\mathrm{MOE}=\frac{F\times a\times l^{2}}{8\times f\times J}\;$$

where F is the force, N; a is the distance from the applied force to the support, mm; l is the length of the deflection measuring section, mm; f is the level of deformation, mm; and J is the moment of inertia, mm^4^.

SYLVAC callipers with a measurement accuracy of 0.01 mm were used to determine the sawn timber section (thickness and width). To determine the length, a linear gauge with 1 mm graduation was used. Weight was evaluated using the Radwag tensometer scale. Moreover, the moisture content of each piece was determined using a Tanel HIT-1 hammer moisture meter.

Since the moisture of the timber during the test differed considerably from 12% (between 10.2% and 13.5%), the outcomes were recalculated using Bauschinger’s Equation (2):

(2) $$E_{12}\;=\;E_{MC}\left[1+\alpha_{MC}\times \left(MC-12\right)\right]$$

where *E*_12_ is the modulus of elasticity of wood for a moisture content of 12%, N/mm^2^; *E_MC_* is the modulus of elasticity of wood for a moisture content of 4% < w < 20%; *α_MC_* is the coefficient of variation of the modulus of elasticity of wood after its moisture content changed by 1%, which is assumed to be 0.02; and *MC* is the absolute moisture content of wood, %.

The assessment covered 486 timber items (samples). Each piece was given an individual number so that it was easy to prepare the lumber sets for the production of beams.

The results of the experimental measurements were analyzed using the STATISTICA 13.0 package (Version 13.0, StatSoft Inc., Tulsa, OK, USA). The obtained results were evaluated using ANOVA (analysis of variance) variance analysis, whereas Tukey’s test was most frequently used to determine homogeneous groups.

## 3. Results and Discussion

**Figure 3:**
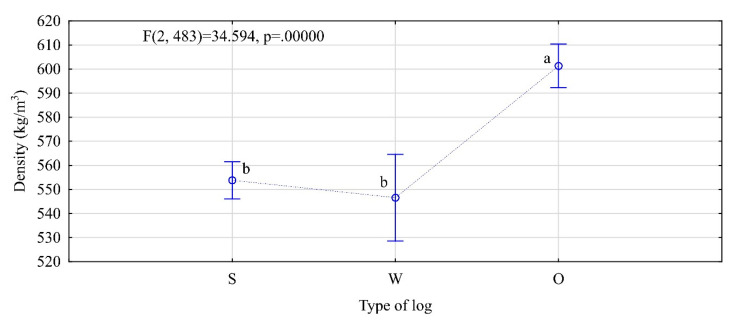
Density of analyzed timber (W—top logs, S—middle logs, O—butt-end logs. The letters denote uniform groups, as determined by Tukey’s HSD (honest significant difference) test).

In the evaluation of structural timber, density is one of the crucial or most important parameters of interest. Average density of the timber items was 571 kg/m^3^ (average moisture 8.98%). The items obtained from the top and middle logs were characterized by similar densities. For those from the butt-end logs, the density was much higher (50 kg/m^3^) (Figure 3).

**Figure 4:**
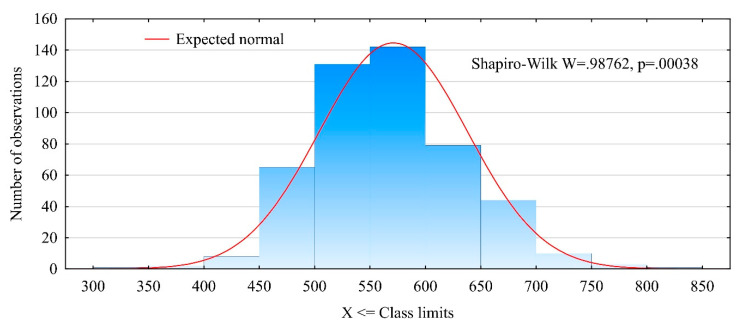
Density distribution for the timber.

The tested timber did not follow a normal density distribution (Figure 4). Its density distribution was characterized by a skewness of 0.343 and kurtosis of 0.361. Hence, the timber’s density distribution had a positive skew, which means that the most of analyzed timber items were characterized by densities below 600 kg/m^3^. This was confirmed by previous observations and data concerning the number of items originating from the various locations along the tree length. It should be noted that the number of butt-end logs was half that of the middle logs.

**Figure 5:**
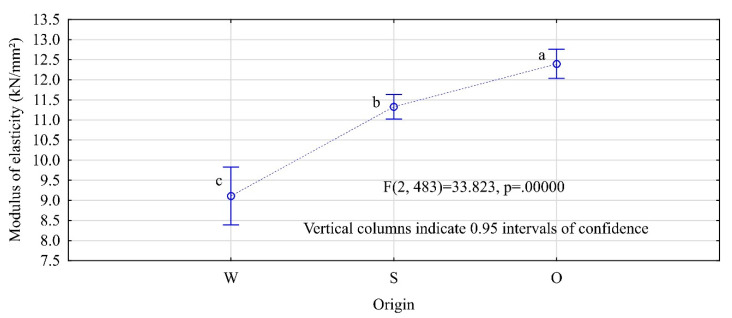
MOE of timber depending on the location of the log along the tree length (W—top logs, S—middle logs, O—butt-end logs. The letters denote uniform groups, as determined by Tukey’s HSD test).

When analyzing density in accordance with EN 338 as an indicator of the strength class of coniferous timber, classes C24, C27, and C30 would comprise only one timber item each, C45 with nine items and C50 the remaining ones. This means that for this batch of timber, its strength is determined mainly by its modulus of elasticity. Statistically, significant differences between the moduli of elasticity were observed for timber items located in different sections along the tree length. The highest moduli were observed for the butt-end logs and the lowest or the top logs. In the first case, the mean value of the modulus of elasticity was 12.4 kN/mm^2^ and in the second was 9.10 kN/mm^2^. The middle-section timber items were characterized by an average modulus of elasticity of 11.3 kN/mm^2^ (Figure 5). Timber from the top section represented classes below C20, timber from the middle section classes C24–C27, and the butt-end timber represents classes above C30. What is important, even though the adopted model of assessing the modulus of elasticity is very different from the assumptions of EN-408, it does provide values close to those stated in that standard [29]. A study has shown that the average modulus of elasticity of timber from the region of interest was 11,915 N/mm^2^ for a SD (standard deviation) = 2440 N/mm^2^, whereas in the test sample of timber, the average modulus was around 11,556 N/mm^2^ for a SD of 2554 N/mm^2^. This indicates that the adopted method of evaluation of the modulus of elasticity in this case provided convergent results for the modulus of elasticity.

**Figure 6:**
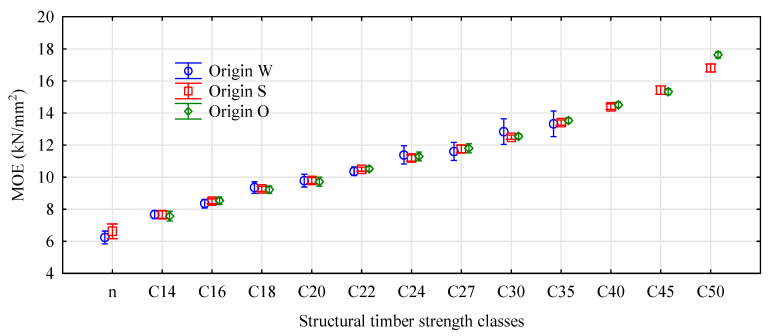
Timber strength classes by location. Legend: W—top logs, S—middle logs, O—butt-end logs.

A more detailed analysis of the distribution of MOE values for the timber items of interest is illustrated in Figure 6.

The broadest range was represented by the timber obtained by sawing the mid-section logs, which comprised both items classified as class C50 and classes below C14. The butt-end log timber, even though its modulus of elasticity was very high, comprised items of high average strength classes ranging from C14 to C50. The top log timber was represented both by unclassified items and those graded between C14 and C35.

**Figure 7:**
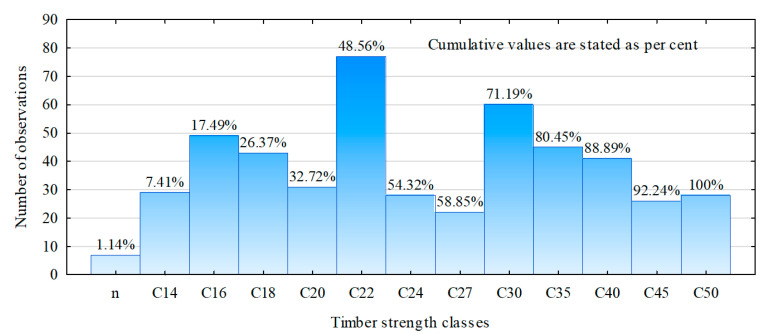
Number of items in strength classes for timber from the Forest Division Olesno.

Figure 7 shows the number of items representing the respective classes of timber strength and the cumulative percentage of the number of unclassified timber items relative to class C50.

C24 is the timber class that is essentially used for roof truss systems and other load-bearing components. This class was represented by a rather low number of items, merely 5.7% of the analyzed timber items. On the other hand, there were only 48.6% of timber items below class C24. The region thus provides timber with exceptionally high mechanical strength, especially with regard to the parameter of interest, modulus of elasticity, which is loosely correlated with bending strength. Since the importance of the quality of timber, in this case, its modulus of elasticity, increases with a squared distance between the timber item’s axis and the beam’s neutral axis, then the higher the beam, the lower the grade of the timber that may be positioned nearer the neutral axis. This means that the higher the beam, the higher number of low grades of timber can be compensated with good grade timber positioned nearer the outer beams. Therefore, the authors chose to prepare such technical models of beams as could be obtained from the available timber.

## 4. Conclusions

The origin of the logs from the trunk (*Pinus sylvestris* L.) has a significant impact on the physical and mechanical properties of the wood from which they are made. Only butt-end logs (log type O) allows for the production of high-quality timber elements.

The pine timber from *Pinus sylvestris* L., which was evaluated in this paper, had a high density of about 570 kg/m^3^ and a high percentage of timber items assigned to class C24 and higher (above 50%), as found in the 4-point bending test to assess its modulus of elasticity.

We used the horizontal model of evaluation of the modulus of elasticity of which the results, for the batch of interest, were similar to those obtained in an evaluation according to EN-408 [25,28].
